# Tailoring the Two Dimensional Electron Gas at Polar ABO_3_/SrTiO_3_ Interfaces for Oxide Electronics

**DOI:** 10.1038/srep13314

**Published:** 2015-08-26

**Authors:** Changjian Li, Zhiqi Liu, Weiming Lü, Xiao Renshaw Wang, Anil Annadi, Zhen Huang, Shengwei Zeng, Ariando, T. Venkatesan

**Affiliations:** 1 NUSNNI-Nanocore, National University of Singapore, Singapore 117411, Singapore; 2 National University of Singapore Graduate School for Integrative Sciences and Engineering (NGS), 28 Medical Drive, Singapore 117456; 3 Department of Physics, National University of Singapore, Singapore 117542, Singapore; 4 Department of Electrical and Computer Engineering, National University of Singapore 117556, Singapore; 5 Department of Materials Science & Engineering, National University of Singapore 117575, Singapore

## Abstract

The 2D electron gas at the polar/non-polar oxide interface has become an important platform for several novel oxide electronic devices. In this paper, the transport properties of a wide range of polar perovskite oxide ABO_3_/SrTiO_3_ (STO) interfaces, where ABO_3_ includes LaAlO_3_, PrAlO_3_, NdAlO_3_, NdGaO_3_ and LaGaO_3_ in both crystalline and amorphous forms, were investigated. A robust 4 unit cell (uc) critical thickness for metal insulator transition was observed for crystalline polar layer/STO interface while the critical thickness for amorphous ones was strongly dependent on the B site atom and its oxygen affinity. For the crystalline interfaces, a sharp transition to the metallic state (i.e. polarization catastrophe induced 2D electron gas only) occurs at a growth temperature of 515 °C which corresponds to a critical relative crystallinity of ~70 ± 10% of the LaAlO_3_ overlayer. This temperature is generally lower than the metal silicide formation temperature and thus offers a route to integrate oxide heterojunction based devices on silicon.

The two dimensional electron gas (2DEG) between two oxide insulators LaAlO_3_ (LAO) and SrTiO_3_ (STO)^1^ has been an active research area on account of the interesting physics at these interfaces such as field tunable metal insulator transition^2^, 2D superconductivity^3^, magnetic interaction^4^, coexistence of superconductivity and ferromagnetism^5^,^6^,^7^. These properties not seen in bulk but arise at the LAO/STO interfaces indicates that electronic/orbital reconstruction plays a crucial role. The proposed polarization catastrophe model^8^, describing electronic reconstruction at the LAO/STO interface to prevent the electrostatic potential divergence with LAO thickness, fits well in the picture. The polarization catastrophe model lead to extensive reports on conductive crystalline ABO_3_/STO heterostructures, including NdGaO_3_, NdAlO_3_, PrAlO_3_, etc^9^,^10^,^11^,^12^. The model was challenged when Chen *et al.*^13^ reported 2DEG with similar carrier concentration and mobility at amorphous LAO/STO interface. It was also found that the conductivity of crystalline LAO/STO interface was also affected by oxygen vacancies created during the growth process^14^. Our prior work^15^ classified the 2DEG into two different carriers at LAO/STO interface: one from polar catastrophe (2DEG-P) and the other from oxygen vacancies in STO (2DEG-V). It was shown that the two carriers had different activation energies, 2DEG-P (~0.5 meV) and 2DEG-V (~4 meV) which lead to the 2DEG-V showing carrier freeze-out at low temperature while the 2DEG-P remained degenerate down to 2 K. However, the comparison between 2DEG-P and 2DEG-V in diverse ABO_3_/STO interfaces has not been explored yet as this could be important for oxide electronic devices. For instance, the amorphous oxide with low oxygen affinity does not lead to any conductivity at the amorphous ABO_3_/STO interface. Will such polar oxide overlayers generate only 2DEG-P at the crystalline interface thereby increasing device reproducibility? What about the degree of crystallinity of the polar layer required for producing a 2DEG-P? We have shown the importance of crystallinity for 2DEG-P formation in our prior work^15^. In addition, Mathew *et al.*^16^ reported that ion beam irradiation induced defects reduce carrier conductivity of LAO/STO interface and can be used for patterning 2DEG-P. Hence, determination of the critical crystallinity for stable 2DEG-P is very important especially for future oxide electronics applications. For example, 2D electron gas at complex oxide interfaces is unique as the entire medium is transparent in the visible to mid-IR suggesting the possibility of opto-electronic devices based on this interface. Furthermore, as the carrier density can be tuned by the back gate voltage in virtue of the large dielectric constant of SrTiO_3_, this could enable optical tunable properties as well. For wide spread application of oxide electronics, the compatibility for oxide growth on silicon is important, however silicon reacts aggressively with most metals at typical film growth temperature about 600 °C^17^. Thus a systematic study of the temperature growth window of polar/non-polar interface is highly desired. Here we address these issues in various ABO_3_/STO interfaces by a systematic comparison of transport properties of various amorphous and crystalline interfaces, including a growth temperature dependent study.

## Experimental

All the samples were prepared by pulsed laser deposition equipped with in-situ reflection high energy electron diffraction (RHEED) with the KrF excimer laser (λ = 248 nm). Laser energy density and frequency was kept at 1.2 J/cm^2^ and 1 Hz for all depositions. Prior to deposition, (001) STO substrates were buffered-HF treated and air annealed in order to get TiO_2_ terminated atomically flat surfaces. Crystalline ABO_3_/STO heterostructures were deposited at 750 °C and amorphous ones at room temperature ~25 °C. (The temperature was measured by a thermocouple spot welded to the heater plate surface. While the actual temperature of the substrate surface could be smaller by as much as 50 °C, as it is difficult to measure hence we quote here only the heater surface temperatures). LAO/STO samples with different crystallinity were deposited at different temperatures from 25 °C to 850 °C and characterized both by X-ray diffraction (XRD) and transport measurements. Oxygen pressure for all depositions was kept at 10^−2^ Torr. The thickness of crystalline films was monitored by RHEED oscillation, for amorphous samples this was controlled by growth duration. After deposition and cooling in deposition atmosphere, samples are named as as-deposited samples, and samples went through additional oxygen annealing process (at 500 °C in flowing 0.5 bar oxygen for two hours) are referred as oxygen annealed sample. All amorphous interfaces become insulting after the additional oxygen annealing steps. Transport properties of amorphous interfaces thus were carried out on as-deposited samples. Transport measurements were performed by Quantum Design Physical Property Measurement System (PPMS), resistance and Hall measurement was measured by Van der Pauw configurations with ultrasonic Al bonded contacts.

## Results and Discussion

**Figure 1:**
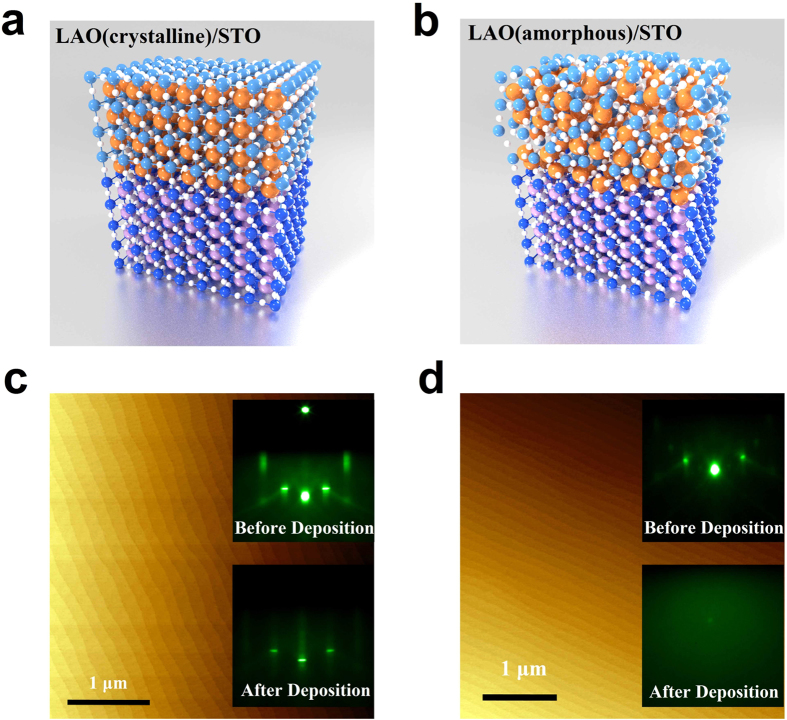
Schematic images of (a) crystalline and (b) amorphous LAO/STO heterojunction. AFM images of topography of (**a**) crystalline and (**b**) amorphous LAO/STO heterojunctions, respectively. Inset of (**c**,**d**), RHEED patterns of LAO/STO before and after LAO deposition.

Figure 1a,b show the schematic images of the crystalline and amorphous LAO/STO interfaces, respectively. The distinct difference between these two interfaces is the absence of the long-range order in the amorphous interfaces. The prerequisite for the polar catastrophe is the alternative stacking of positive and negative charge sheets of the polar layer such that a 2DEG-P can be formed when the build-up potential is larger than the bandgap of STO. Clearly amorphous interface does not satisfy that condition. Experimentally, crystalline and amorphous heterojunctions show very similar topography in atomic force microscopy (AFM) images. As shown in Fig. 1c,d, both surfaces show atomic terraces which resemble the surface of the treated STO substrate surface. Nonetheless, the crystallinity can be detected by RHEED pattern during the deposition. RHEED patterns before and after deposition of crystalline and amorphous LAO/STO heterojunctions are shown in insets of Fig. 1c,d. After deposition, a streaky pattern is observed in crystalline LAO deposition while a faint uniform background in amorphous film. The diffuse scattering dominates over coherent scattering due to lack of lattice periodicity at the amorphous LAO film surface so that there is no observable line or dot pattern.

**Figure 2:**
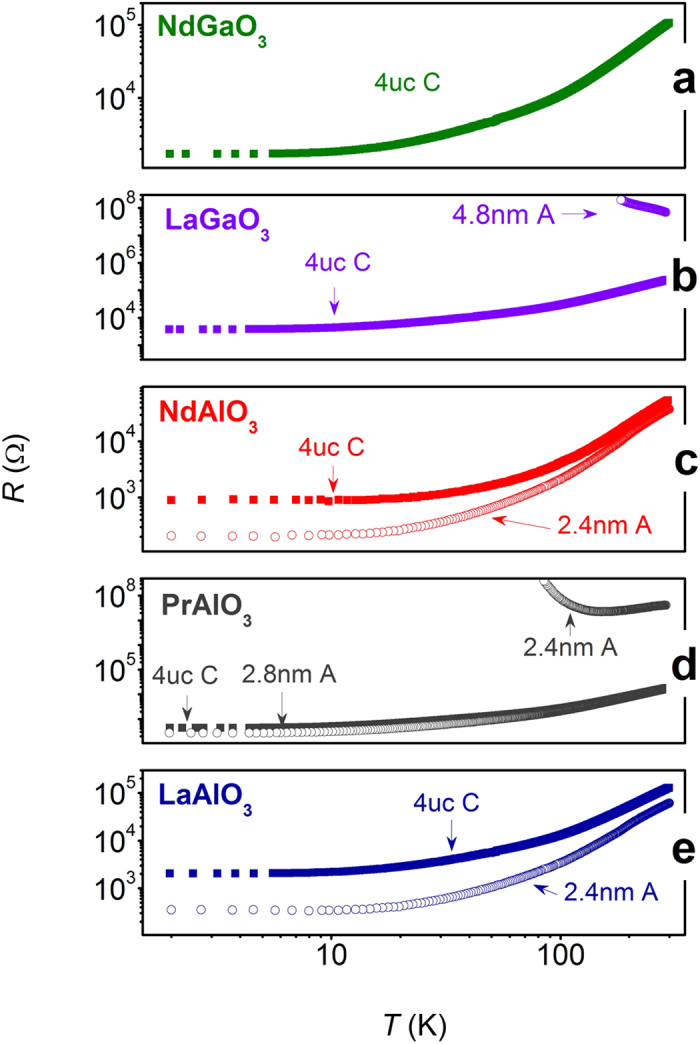
Temperature dependent sheet resistance for crystalline and amorphous ABO_3_/STO with different thicknesses of ABO_3_- (**a**) NdGaO_3_, (**b**) LaGaO_3_, (**c**) NdAlO_3_, (**d**) PrAlO_3_ and (**e**) LaAlO_3_. “4 uc C” means 4 uc (~1.6 nm) of polar ABO_3_ layer in crystalline form, and “2.4 nm A” refers to 2.4 nm of polar layer in amorphous form.

Sheet resistances versus temperature of crystalline and amorphous NdGaO_3_ (NGO), PrAlO_3_ (PAO), NdAlO_3_ (NAO) and LAO /STO interfaces are summarized in Fig. 2a–e, respectively. It is found that all 4 uc oxygen annealed crystalline interfaces show metallic behavior in the whole temperature regime from 300 to 2 K. However variations are seen in amorphous interfaces. Primarily, amorphous heterostructure seems to be strongly affected by B-site element, i.e. AAlO_3_ (aluminate) based interface is more conductive than AGaO_3_ (gallate) based interface. Amorphous NGO/STO interface shows no measurable conductivity even when the thickness of NGO is up to 5 nm. In addition, we have observed a transition from semiconducting to metallic behavior in amorphous PAO/STO heterojunction with increasing PAO thickness from 2.4 to 2.8 nm (Fig. 2d), which illustrates how resistivity behavior is affected across the Mott limit by increasing amount of oxygen vacancies. This B-site element dependent conductivity of polar/non-polar interface strongly suggests that the elemental chemical redox reaction model is dominating in the amorphous case. It is consistent with the fact that aluminum oxygen affinity is much stronger than that of gallium^18^.

**Figure 3:**
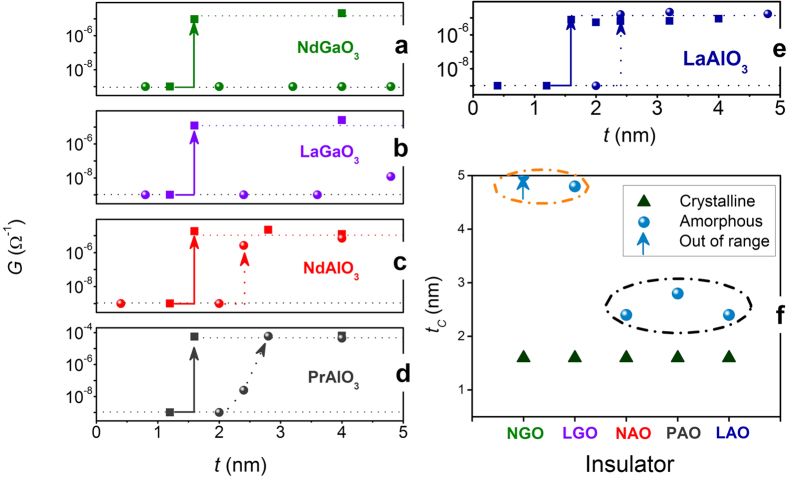
Thickness dependent conductance of crystalline (solid squares) and amorphous (solid spheres) ABO_3_/STO where ABO_3_ includes (a) NdGaO_3_, (b) LaGaO_3_, (c) NdAlO_3_, (d) PrAlO_3_ and (e) LaAlO_3_. Crystalline ABO_3_/STO show universal critical thickness of 4 uc while critical thickness of amorphous ABO_3_/STO is dependent on B-site atoms, as shown in (**f**). The solid arrow represents the metal insulator transition for crystalline interfaces while dashed arrow for amorphous interfaces transition. Dash lines are given as guides to the eye.

The other difference between crystalline and amorphous ABO_3_/STO is on critical thickness for metal insulator transition. Figure 3a–e show the room temperature conductivity versus ABO_3_ layer thicknesses in both amorphous and crystalline interfaces. In crystalline interfaces, all interfaces show a sharp metal insulator transition occurring at 4 uc where 4 orders (10^−9^ Ω^−1^ to 10^−5^ Ω^−1^) of conductance change was observed at room temperature by adding only one unit cell from 3 uc, consistent with earlier results^10^. Although aluminate and gallate introduce diverse interfacial strain and local chemical environment, the polar nature of the overlayer dominates the creation of 2DEG-P process. In contrast, the critical thickness for the conducting amorphous ABO_3_/STO is more dependent on the oxygen affinity of B-site cation besides experimental conditions (e.g. oxygen pressure). As can be seen in Fig. 3a–e and summarized in 3f, critical thicknesses of amorphous AAlO_3_/STO interfaces are between 2.4 to 2.8 nm, much smaller than AGaO_3_/STO interfaces (≥5 nm).

**Figure 4:**
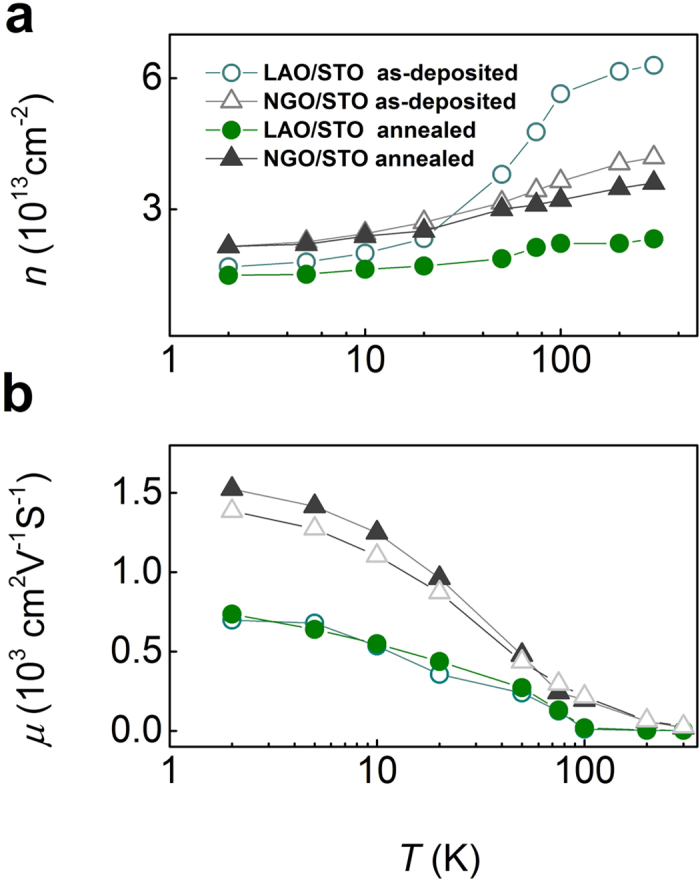
(**a**) Carrier density and (**b**) carrier mobility versus temperature curves for 4 uc LAO/STO and NGO/STO interface before and after oxygen annealing, respectively.

The difference in oxygen vacancy creation between aluminate and gallate polar oxide also affects the transport properties of crystalline interfaces. In Fig. 4a, carrier density is more temperature sensitive in 4 uc as deposited crystalline LAO/STO interface than the counterpart NGO/STO interface; similar to previous report^19^. Moreover, oxygen annealing has less effect in temperature dependent carrier density curve of NGO/STO interface. This direct evidence confirms that the oxygen vacancy content in 4 uc NGO/STO interface is much less compared to LAO/STO interface. The other noticeable trend is that carrier mobility in NGO/STO interface is higher than that in LAO/STO interface (see Fig. 4b) as NGO/STO has less strain compared with LAO/STO (1.1% versus 3.0% in LAO/STO). The key point here is that by using a less oxygen affinity B cation the 2DEG in the crystalline case is dominantly from polar contribution which will enhance the reproducibility of devices as they will be less affected by oxygen pressure during processing steps.

**Figure 5:**
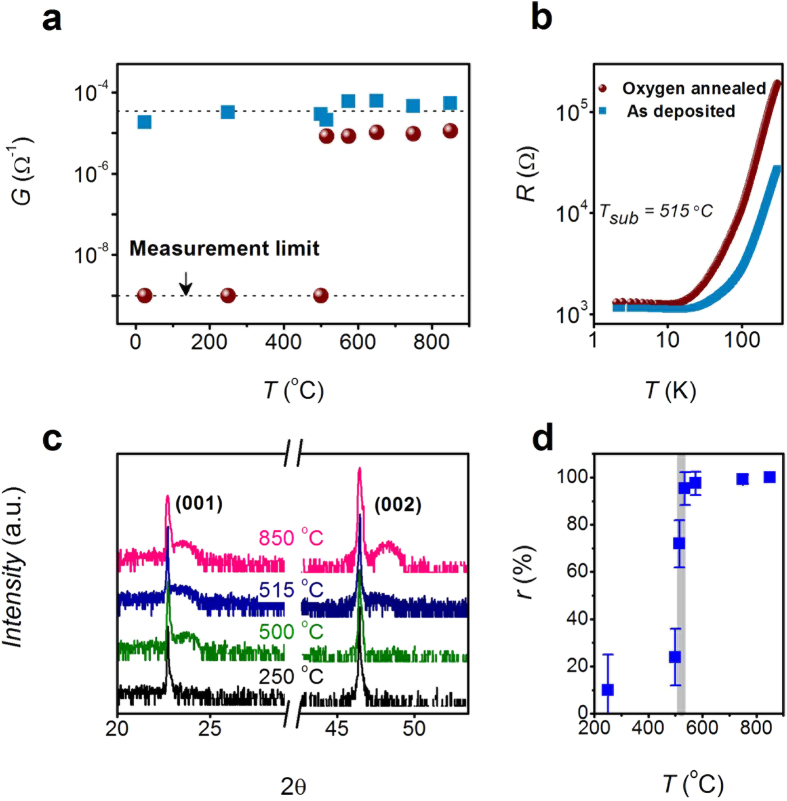
(**a**) Deposition temperature dependence of room temperature conductance of as deposited (light blue squares) and oxygen annealed (dark red spheres) LAO/STO interfaces with LAO thickness of 4 nm (10 uc). (**b**) Temperature dependence of resistance of as deposited and oxygen annealed LAO/STO prepared at 515 °C. (**b**) X-ray diffraction (XRD) spectra of LAO/STO heterojunction with fabrication temperature of 250, 500, 515 and 850 °C. (**c**) Growth temperature dependence of normalized crystallinity, r, defined as area ratio of (002) LAO to STO XRD peak normalized to area ratio at growth temperature of 850 °C. The solid line in (**d**) is given as guide to the eye.

To find the critical crystallinity for a stable 2DEG-P, we did a systematic study on LAO/STO interfaces prepared at different substrate temperatures from 25 to 850 °C. The thickness of LAO was kept at 4 nm (10 uc). As shown in Fig. 5a, all the as-deposited samples (light blue squares) are conducting with conductance ~2 × 10^−5^ Ω^−1^ at room temperature and show metallic behavior without significant difference. After oxygen annealing at 500 °C with 0.5 bar of oxygen flow, samples (dark red solid spheres) prepared at 500 °C and below become highly insulating while samples prepared at 515 °C and above are still conducting with about seven fold increase of sheet resistance (see Fig. 5b). After oxygen annealing, 2DEG-V is removed, 2DEG-P is the only responsible mechanism for the metallic behavior. As mentioned before, high crystallinity is crucial for 2DEG-P. The critical temperature of 515 °C is most likely the crystallization temperature of LAO film.

This is confirmed by the XRD spectra in Fig. 5c where no distinct LAO XRD peak is seen when the substrate temperature is below 250 C. LAO peaks start to appear when the substrate temperature is above 500 °C and saturate at 600 °C and above. Due to the difficulty of quantifying crystallinity precisely, we use the area ratio of LAO (200) peak to substrate STO (200) peak as a figure of merit. The area ratio is normalized to the area ratio at 850 °C. Figure 5d shows the area ratio, r in percentage, dependence on the substrate temperature. A sharp increase occurs at ~500 °C. Within a narrow window from 500 to 575 °C, the crystallinity increases from 20 ± 15% to nearly 100% rapidly, which qualitatively describes the crystallization near the crystallization temperature. The minimum temperature for stable 2DEG-P of 515 °C corresponds to the area ratio of ~70 ± 10%, which defines the critical crystallinity for 2DEG-P formation in LAO/STO system. The incompletely crystalized LAO film can be divided into crystalline and amorphous region. Crystalline regions induce electron transfer and form localized conductive islands while area under amorphous region is insulating. When the conductive regions reach the 2D percolation threshold, a macroscopic conducting network is established which shows global conductivity. This picture is evidenced by the fact that carrier density of 2DEG-P is lower at low growth temperatures^20^. This is analogues to our previous result that ~65% surface coverage of 4^th^ uc of LAO is the threshold value for metal-insulator-transition in crystalline LAO/STO interface^21^. Fortuitously, the minimum growth temperature of 515 °C is just adequate to avoid silicide formation (e.g. TiSi_2_ and SiSr_2_ formation at the STO/Si interface at 600–700^17^ and 650–700 °C^22^,^23^, respectively.), which accounts for structural quality degradation of epitaxial STO on Si. As a result, 2DEG-P properties at ABO_3_/STO interface are ready for integration with silicon considering that a number of groups have reported on high quality epitaxial STO growth on Si^24^,^25^,^26^,^27^,^28^.

## Conclusion

In summary, through systematic comparison between transport properties for a series of polar ABO_3_/STO interfaces, we have shown that the relative contribution of 2DEG-P and 2DEG-V to the transport can be tailored by varying the B cation. In addition, critical substrate temperature of 515 °C marks the onset of 2DEG-P formation at the LAO/STO interface which corresponds to a crystallinity of 70 ± 10%. These results offer alternate experimental evidences for the requirement of an ordered polar layer for the polar catastrophe model at a crystalline interface and the oxygen redox model at an amorphous interface. Further, the work sheds light on the role of key process parameters (temperature and oxygen pressure during growth) for the possible integration of oxide heterojunction based devices on silicon.

## Additional Information

**How to cite this article**: Li, C. *et al.* Tailoring the Two Dimensional Electron Gas at Polar ABO_3_/SrTiO_3_ Interfaces for Oxide Electronics. *Sci. Rep.* **5**, 13314; doi: 10.1038/srep13314 (2015).
